# Risk Factors for Nonalcoholic Fatty Liver Disease with Different Insulin Resistance in a Nonobese Chinese Population

**DOI:** 10.1155/2022/9060405

**Published:** 2022-12-14

**Authors:** Xiaojuan Wu, Ying Wang, Yumei Jia, Jia Liu, Guang Wang

**Affiliations:** ^1^Department of Endocrinology, Beijing Chaoyang Hospital, Capital Medical University, No. 8, Gongti South Road, Chaoyang District, Beijing 100020, China; ^2^Department of Medical Center, Beijing Chaoyang Hospital, Capital Medical University, No. 8, Gongti South Road, Chaoyang District, Beijing 100020, China

## Abstract

**Purposes:**

The aim of this study is to identify the risk factors of nonobese nonalcoholic fatty liver disease (NAFLD) individuals under different insulin resistance status.

**Methods:**

This cross-sectional study was conducted at the Medical Center of Beijing Chaoyang Hospital affiliated with Capital Medical University. NAFLD was diagnosed based upon ultrasonographic findings consistent with fatty liver disease.

**Results:**

A total of 1257 nonobese adults (625 non-NAFLD and 632 nonobese NAFLD) with body mass index (BMI) 18.5-24.9 kg/m^2^ were enrolled in the study. And all patients were divided into homeostasis model assessment of insulin resistance (HOMA − IR) > 1 group and HOMA − IR ≤ 1 group. When all the variables were adjusted in both the HOMA − IR > 1 group and HOMA − IR ≤ 1 group, older age (>50 years), higher BMI (23.0-24.9 kg/m^2^), higher AST (>18 U/L), higher TG (>0.9 mmol/L), higher GLU (>5.25 mmol/L), and higher HbA1C (>5.5%) were associated with higher risks of nonobese NAFLD. In patients with HOMA − IR > 1, lower homeostatic model assessment of *β*-cell function (HOMA-*β*) (<47.1%) (OR, 7.460, 95% CI, 3.051-18.238, *P* < 0.001) was associated with higher risks of nonobese NAFLD.

**Conclusion:**

s. Metabolic profiles (i.e., higher BMI, hyperglycemia, hypertriglyceridemia, and higher glycosylated hemoglobin) are risk factors of nonobese NAFLD, regardless of insulin resistance status. Decreased function of pancreatic *β*-cells may be the risk factor of nonobese NAFLD with insulin resistance, who should pay attention to further development of pancreatic *β*-cell dysfunction.

## 1. Introduction

The prevalence of nonalcoholic fatty liver disease (NAFLD) increased from 25.28% to 29.62% between 1999 and 2019 [1]. Obesity is a key risk factor associated with NAFLD incidence, but rates of NAFLD among nonobese individuals are also rising. Around 8-19% of Asians with body mass indexes less than 25 kg/m^2^ are found to have NAFLD, a condition often described as “lean” or “nonobese” NAFLD [2]. There is some evidence that nonobese NAFLD patients may exhibit poorer outcomes than obese NAFLD patients, developing cirrhosis at a more rapid rate [3]. Insulin resistance is one of the key factors implicated in the development and progression of NAFLD [4]. However, the risk factors under different insulin resistance status of nonobese NAFLD remain unclear. The present study was to identify the risk factors under different insulin resistance status of nonobese NAFLD.

## 2. Methods

### 2.1. Population

This was a cross-sectional study conducted from November 2018 to October 2019 at the Medical Center in Beijing Chaoyang Hospital affiliated with Capital Medical University. We consecutively recruited 1257 nonobese subjects. Inclusion criteria were as follows: (1) age ≥ 18 years, (2) BMI 18.5-24.9 kg/m^2^ [5], and (3) 3.9 mmol/L < fasting blood glucose (FBG) < 6.1 mmol/L [6]. Patients were excluded if they exhibited (1) abnormal liver function: more than the upper limit of normal value (alanine aminotransferase (ALT) > 50 U/L or aspartate aminotransferase (AST) > 40 U/L); (2) BMI < 18.5 or ≥ 25 kg/m^2^; (3) systolic blood pressure ≥ 140 mmHg; (4) diastolic blood pressure ≥ 90 mmHg; (5) significant alcohol intake (>70 or >140 g/week for women and men, respectively); (6) liver disease associated with other conditions including viral hepatitis, autoimmune hepatitis, hemochromatosis, or Wilson's disease; and (7) fatty liver associated with other conditions such as drug use or hereditary disease. Participants provided written informed consent, and the Ethics Committee of the Beijing Chaoyang Hospital, Capital Medical University, approved the present study.

### 2.2. Measurements

The general health checkup included a medical history, physical examination, lifestyle questionnaire, biochemical measurements, and abdominal ultrasonography. Blood specimens were sampled from the antecubital vein after more than 12 h of fasting. Colorimetric assays were used to measure total cholesterol (TC), low-density lipoprotein cholesterol (LDL-c), high-density lipoprotein cholesterol (HDL-c), and triglyceride (TG) via the use of an autoanalyzer (Hitachi 7170). Fasting blood glucose (FBG), fasting insulin (FINS), and HbA1c levels were analyzed at the Central Chemistry Laboratory of Beijing Chaoyang Hospital affiliated with Capital Medical University. BMI was calculated as *weight* (*kg*)/*height* (*m*^2^). The homeostasis model assessment of insulin resistance (HOMA-IR) index was calculated according to the formula HOMA‐IR = (FINS(uIU/mL) × FBG(mmol/L))/22.5. Homeostatic model assessment of *β*-cell function (HOMA-*β*) index was calculated according to the formula HOMA‐*β* = (20 × FINS(uIU/mL))/(FBG(mmol/L)‐3.5) [7].

Abdominal ultrasounds were performed by three experienced radiologists who were unaware of the purpose of the study and blinded to laboratory values. NAFLD was diagnosed as per the criteria of the Chinese Liver Disease Association [8]. Fatty liver was defined by the presence of diffuse enhancement of near-field echo in the hepatic region and gradual attenuation of the far-field echo in combination with an unclear intrahepatic lacuna structure, mild-to-moderate hepatomegaly with a rounded or blunt border, or color Doppler ultrasonography revealing reduced hepatic blood flow with a normal blood flow distribution [9].

### 2.3. Statistical Methods

SPSS 22.0 (SPSS Inc., IL, USA) was employed for statistical analyses. The differences of covariates between the NAFLD status were compared using *t*-test or the Wilcoxon signed-rank test for continuous variables and chi-square test for categorical variables. Collinearity diagnostic was conducted prior to further assessment. A stepwise multivariable logistic regression was performed (backward: Wald; entry: 0.05; removal: 0.10) to identify risk factors linked with nonobese NAFLD. *P* values of <0.05 were considered statistically significant.

## 3. Results

### 3.1. Baseline Patient Characteristics

Of the 1257 nonobese subjects aged 18–79 years, there were 625 non-NAFLD and 632 nonobese NAFLD. In all subjects, 877(69.8%) were men (Table 1). Subjects with nonobese NAFLD were more likely to be older and middle aged (31-50years) (*P* < 0.001), higher BMI (*P* < 0.001), higher ALT (*P* < 0.001), higher AST (*P* < 0.001), higher TG (*P* < 0.001), higher LDL-c (*P* = 0.031), higher fasting glucose (*P* < 0.001), higher insulin (*P* < 0.001), and higher HbA1c (*P* < 0.001) values relative to patients without NAFLD. However, subjects with NAFLD were more likely to have lower HDL-c (*P* < 0.001) and lower HOMA-*β* (*P* < 0.001) (Table 1).

**Figure 1:**
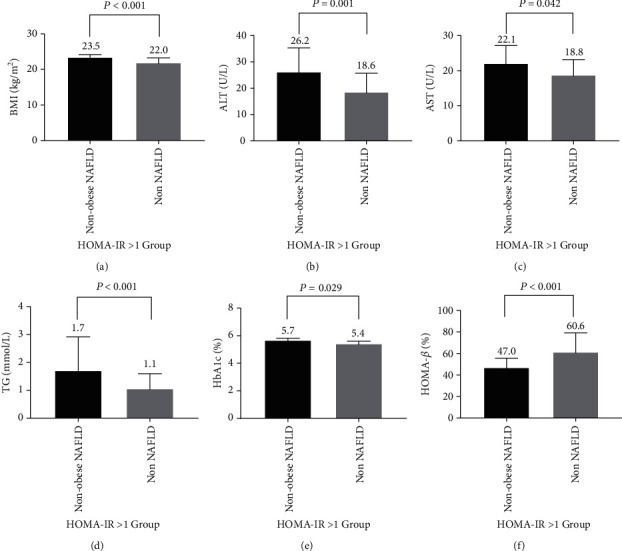
HOMA − IR > 1 group. The characteristics of the participants of nonobese NAFLD and non-NAFLD. (a) BMI; (b) ALT; (c) AST; (d) TG; (e) HbA1c; (f) HOMA-*β*.

In the HOMA − IR > 1 group, the subjects with nonobese NAFLD also had higher BMI (23.5 ± 1.0 vs. 22.0 ± 1.6 kg/m^2^, *P* < 0.001) (Figure 1(a)), higher ALT (26.2 ± 9.5 vs. 18.6 ± 7.6 U/L, *P* = 0.001) (Figure 1(b)), higher AST (22.1 ± 5.4 vs. 18.8 ± 4.6 U/L, *P* = 0.042) (Figure 1(c)), higher TG (1.7 ± 1.2 vs. 1.1 ± 0.6 mmol/L, *P* < 0.001) (Figure 1(d)), and higher HbA1c (5.7 ± 0.2 vs. 5.4 ± 0.3%, *P* = 0.029) (Figure 1(e)) values relative to patients without NAFLD. The subjects with nonobese NAFLD had lower HOMA-*β* (47.0 ± 9.7 vs. 60.6 ± 18.7%, *P* < 0.001) compared to those without NAFLD (Figure 1(f)).

**Figure 2:**
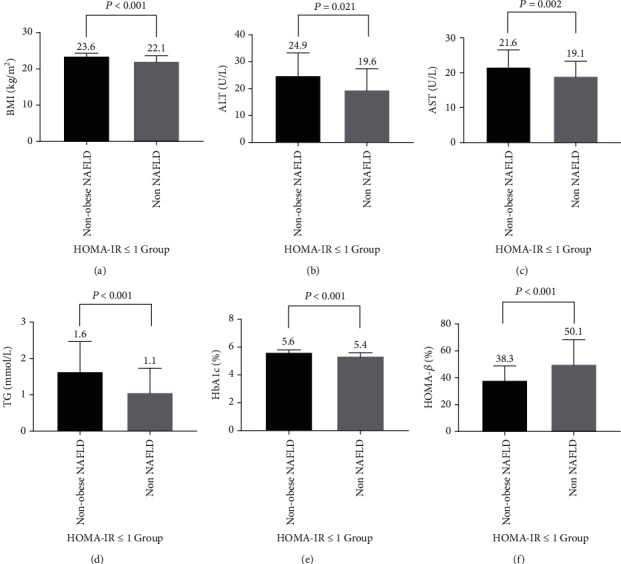
HOMA − IR ≤ 1 group. The characteristics of the participants of nonobese NAFLD and non-NAFLD. (a) BMI; (b) ALT; (c) AST; (d) TG; (e) HbA1c; (f) HOMA-*β*.

In the HOMA − IR ≤ 1 group, the subjects with nonobese NAFLD also had higher BMI (23.6 ± 1.0 vs. 22.1 ± 1.8 kg/m^2^, *P* < 0.001) (Figure 2(a)), higher ALT (24.9 ± 8.8 vs. 19.6 ± 8.4 U/L, *P* = 0.021) (Figure 2(b)), higher AST (21.6 ± 5.2 vs. 19.1 ± 4.6 U/L, *P* = 0.002) (Figure 2(c)), higher TG (1.6 ± 0.8 vs. 1.1 ± 0.7 mmol/L, *P* < 0.001) (Figure 2(d)), and higher HbA1c (5.6 ± 0.2 vs. 5.4 ± 0.3%, *P* < 0.001) (Figure 2(e)) values relative to patients without NAFLD. The subjects with nonobese NAFLD had lower HOMA-*β* (38.3 ± 11.3 vs. 50.1 ± 19.1%, *P* < 0.001) compared to those without NAFLD (Figure 2(f)).

### 3.2. Risk Factors Associated with Nonobese NAFLD

#### 3.2.1. Risk Factors Associated with Nonobese NAFLD in Patients with HOMA − IR > 1

In patients with HOMA − IR > 1, when no variable was adjusted and age and gender adjusted, the results showed that older age (>50years), higher BMI (23.0-24.9 kg/m^2^), higher ALT (>17 U/L), higher AST (>18 U/L), lower HDL-c (<1.36 mmol/L), higher TG (>0.9 mmol/L), higher LDL-c (>2.65 mmol/L), higher GLU (>5.25 mmol/L), higher HbA1C (>5.5%), higher FINS (>3.89 uIU/ml), and lower HOMA-*β* (<47.1%) were independent factors. When all the variables were adjusted, the results showed that older age (>50years) (OR, 3.253, 95% CI, 1.224-8.647, *P* = 0.018), higher BMI (23.0-24.9 kg/m^2^) (OR, 5.532, 95% CI, 2.830-10.813, *P* < 0.001), higher AST (>18 U/L) (OR, 2.326, 95% CI, 1.192-4.539, *P* = 0.013), higher TG (>0.9 mmol/L) (OR, 6.069, 95% CI, 2.899-12.708, *P* < 0.001), higher GLU (>5.25 mmol/L) (OR, 2.953, 95% CI, 1.376-6.339, *P* < 0.001), higher HbA1C (>5.5%) (OR, 5.434, 95% CI, 2.765-10.682, *P* < 0.001), higher FINS (>3.89 uIU/ml) (OR, 2.890, 95% CI, 1.492-5.598, *P* = 0.002), and lower HOMA-*β* (<47.1%) (OR, 7.460, 95% CI, 3.051-18.238, *P* < 0.001) were associated with higher risks of nonobese NAFLD (Table 2).

#### 3.2.2. Risk Factors Associated with Nonobese NAFLD in Patients with HOMA − IR ≤ 1

In patients with HOMA − IR ≤ 1, when no variable was adjusted and age and gender adjusted, the results showed that male, older age (41-50 and >50years), higher BMI (23.0-24.9 kg/m^2^), higher ALT (>17 U/L), higher AST (>18 U/L), lower HDL-c (<1.36 mmol/L), higher TG (>0.9 mmol/L), higher GLU (>5.25 mmol/L), higher HbA1C (>5.5%), and lower HOMA-*β* (<47.1%) were independent factors. When all the variables were adjusted, the results showed that older age (41-50years) (OR, 2.993, 95% CI, 1.744-5.137, *P* < 0.001), age > 50 years (OR, 3.086, 95% CI, 1.810-5.261, *P* < 0.001), higher BMI (23.0-24.9 kg/m^2^) (OR, 4.522, 95% CI, 3.104-6.588, *P* < 0.001), higher AST (>18 U/L) (OR, 1.778, 95% CI, 1.231-2.569, *P* < 0.001), higher TG (>0.9 mmol/L) (OR, 3.423, 95% CI, 2.429-4.823, *P* < 0.001), higher GLU (>5.25 mmol/L), and higher HbA1C (>5.5%) (OR, 4.757, 95% CI, 2.601-5.249, *P* < 0.001) were associated with higher risks of nonobese NAFLD (Table 3).

## 4. Discussion

We conducted this study to identify risk factors of nonobese NAFLD individuals under different insulin resistance status in the Chinese population. Our results revealed that when all the variables were adjusted in both the HOMA − IR > 1 group and HOMA − IR ≤ 1 group, older age (>50years), higher BMI (23.0-24.9 kg/m^2^), higher AST (>18 U/L), higher TG (>0.9 mmol/L), higher GLU (>5.25 mmol/L), and higher HbA1C (>5.5%) were associated with higher risks of nonobese NAFLD. In patients with HOMA > 1, lower HOMA-*β* (<47.1%) (OR, 7.460, 95% CI, 3.051-18.238, *P* < 0.001)was associated with higher risks of nonobese NAFLD.

A systematic review of Ye et al. found that nonobese NAFLD patients exhibited increased fasting blood glucose, cholesterol, and HOMA-IR values relative to patients without NAFLD. And HOMA-IR values differed significantly between nonobese and obese NAFLD patients [10]. Similarly, Musso et al. proposed that nonobese NAFLD is more closely linked to insulin resistance, oxidative stress, and endothelial dysfunction [11]. Sinn et al. also determined that nonobese NAFLD was independently predictive of insulin resistance regardless of other metabolic syndrome symptoms in nonobese nondiabetic patients [12]. Our study revealed that 180 (72.3%) of nonobese NAFLD were HOMA − IR > 1.

HOMA-*β* assesses pancreatic *β*-cell function from basal glucose and insulin concentrations and reflects pancreatic *β*-cell insulin secretion under nonstimulated conditions. The relationship between insulin resistance and NAFLD is well established. However, there are a limited number of studies evaluating pancreatic *β*-cell in NAFLD of Asians. Siddiqui et al. reported that nondiabetic subjects with NAFLD have significant pancreatic *β*-cell dysfunction compared controls. And there was a trend towards a decrease in HOMA-*β* in North American patients with NAFLD and NASH with increasing steatosis grade [13]. Musso et al. revealed that Italy nonobese patients with NASH before glucose intolerance appears to be *β*-cell secretory impairment, who were also more insulin resistant than were the controls [14]. Meanwhile, in our study, the subjects with nonobese NAFLD had lower HOMA-*β* (47.0 ± 9.7 vs. 60.6 ± 18.7%, *P* < 0.001) in the *HOMA* − *IR* > 1 group and lower HOMA-*β* (38.3 ± 11.3 vs. 50.1 ± 19.1, *P* < 0.001) in the *HOMA* − *IR* ≤ 1 group. We found that lower HOMA-*β* (<47.1%) had significant higher risk for nonobese NAFLD (OR, 7.460, 95% CI, 3.051-18.238, *P* < 0.001) in the *HOMA* − *IR* > 1 group. Furthermore, Musso et al. reported that microsomal triglyceride transfer protein- (MTP-) 493G/T polymorphism is associated with pancreatic *β*-cell dysfunction in NASH [15]. Siddiqui et al. revealed that HOMA-*β* of the lean group controls (BMI< 25 kg/m2) was nearly 10% [13]. In addition, Cai et al. demonstrated that 22.4% of 263 Chinese NGT had *HOMA* − *β* ≤ 40.12% [16]. In spite of the *HOMA* − *β* < 47.1% of our study, the participants all had normal glucose. It can be explained by the review of Gastaldelli, who revealed that even in the case of a 50% reduction, i.e., after experimental partial pancreatectomy, normoglycemia can be maintained by relatively small pancreas remnants. The system to compensate for changes a reduction in insulin sensitivity by increasing insulin secretion. T2DM develops when the beta-cell secretory capacity is not sufficient to overcome the insulin resistance of the tissues [17].

It is known that NAFLD is highly associated with the metabolic syndrome [18]. In the entire population, all components of the metabolic syndrome are associated with NAFLD [19]. This study on the nonobese population suggests that increased glucose (>5.25 mmol/L), elevated plasma triglycerides (>0.9 mmol/L), higher Hb1Ac levels (>5.5%), and higher ALT levels (>17 U/L) were related to nonobese NAFLD in both the *HOMA* − *IR* > 1 and *HOMA* − *IR* ≤ 1 groups. This is consistent with the prior findings from Wei, who determined that high BMI, and high HbA1c, and insulin resistance were independently associated with NAFLD in nonobese subject [20]. Chen et al. also found that elevated ALT (>40 U/L) was closely related to NAFLD in nonobese adults [21]. Meanwhile, Ampuero et al. found that unhealthy nonobesity NASH was independently linked with HOMA-IR and ALT [22].

We found that nonobese NAFLD patients were more likely to be higher BMI (23.0-24.9 kg/m^2^) and middle-aged relative to patients without NAFLD. Xu et al. previously found that BMI was a primary risk factor linked to NAFLD incidence in nonobese subjects. Even slight increases in BMI within the normal range can elevate the risk of NAFLD development [23]. Zeng et al. determined that the BMI of overweight (BMI 23-25 kg/m^2^) individuals with and without NAFLD was 27.98 ± 2.84 and 26.69 ± 2.27 kg/m^2^, respectively [24]. In the present study, we found that subjects with a BMI of 23.0-24.9 kg/m^2^ were at a higher risk of nonobese NAFLD (OR, 5.532, *P* < 0.001) in the *HOMA* − *IR* > 1 and (OR, 4.522, *P* < 0.001) in the *HOMA* − *IR* ≤ 1 groups. A study conducted in Japan also found that the prevalence of NAFLD increased gradually with age, with a peak prevalence of 23.3% in the 60-to 69-year age group, which was 3.4 times as high as that in the 30- to 39-year age group [25]. We found that*age* > 50years had higher risk for nonobese NAFLD in the*HOMA* − *IR* > 1(OR, 3.253,*P* = 0.018) and in the*HOMA* − *IR* ≤ 1(OR, 3.086,*P* < 0.001) groups. Moreover, Wei et al. showed that the prevalence of NAFLD among nonobese subjects increases with age [20]. In line with the study of Cho who reported that higher BMIs, HOMA-IR values, ALT levels, hypertriglyceridemia, and hyperuricemia were associated with NAFLD in the nonobese Korean subjects [26].

These studies including ours consistently emphasize that nonobese NAFLD is closely associated with metabolic profiles. And people with insulin resistance and nonobese NAFLD should pay attention to further development of pancreatic *β*-cell dysfunction.

Moreover, lean NAFLD has been associated with PNPLA3 polymorphisms. In particular, the substitution of methionine with isoleucine at the residual 148 would limit the access of the substrate to the catalytic serine in position 47, thus triggering an altered hydrolysis of hepatic triglycerides and a consequent increase in the content of cellular triglycerides. All these changes seem to be not associated with insulin resistance [27–29].

There are multiple limitations to the present study. For one, potential limitations of this study are cross-sectional studies where we are unable to determine the longitudinal relationship between nonobese NAFLD, its histological parameters, and pancreatic *β*-cell over time. Another limitation of the study is that NAFLD was detected via ultrasound rather than via liver biopsy, although this approach is the most convenient and common means of diagnosing this condition. The ultrasound method is not quantified. Thirdly, lean NAFLD has been associated with PNPLA3 polymorphisms. The clinical utility of the polymorphisms involved in NAFLD is in part limited by low diffusion of genotyping methods in the routine clinical diagnostics and high cost [30]. Finally, metabolic syndrome is associated with increased insulin resistance. Visceral adiposity, waist circumference, and sarcopenia have emerged to be stricter than BMI. Thus, BMI maybe hard to evaluate in borderline patients [29]. NAFLD in lean patients is often associated with a series of pathologies such as lipodystrophy, lysosomal acid lipase, and familial hypobetalipoproteinemia [31]. Further research is needed to consider the interference of these diseases.

## 5. Conclusion

In conclusion, we found that metabolic profiles (i.e., higher BMI, hyperglycemia, hypertriglyceridemia, and higher glycosylated hemoglobin) are risk factors of nonobese NAFLD, regardless of insulin resistance status. Decreased function of pancreatic *β*-cells may be the risk factor of nonobese NAFLD with insulin resistance, which should be paid attention to further development of pancreatic *β*-cell dysfunction.

## Figures and Tables

**Table 1:** Distributions of study variables among participants with and without nonobese NAFLD.

Characteristic	All subjects (*n* = 1257)	Non-NAFLD (*n* = 625)		Nonobese NAFLD (*n* = 632)		*P* value
		HOMA-IR>1 (*n* = 135)	HOMA-IR ≤1 (*n* = 490)	HOMA-IR>1 (*n* = 249)	HOMA-IR ≤1 (*n* = 383)	
Gender						0.075
Male, *n* (%)	877 (69.8%)	44 (32.6%)	324 (66.1%)	69 (27.7%)	282 (73.6%)	
Female, *n* (%)	380 (30.2%)	91 (67.4%)	166 (33.9%)	180 (72.3%)	101 (26.4%)	
Age (years), *n* (%)						<0.001
18-30	326 (25.9%)	42 (31.1%)	158 (32.2%)	57 (22.9%)	69 (18.0%)	
31-40	338 (26.9%)	32 (23.7%)	111 (22.7%)	70 (28.1%)	125 (32.6%)	
41-50	368 (29.3%)	32 (23.7%)	109 (22.2%)	85 (34.1%)	142 (37.1%)	
>50	225 (17.9%)	29 (21.5%)	112 (22.9%)	37 (14.9%)	47 (12.3%)	
Body mass index (kg/m^2^), *n* (%)						<0.001
18.5-22.9	557 (44.3%)	96 (71.1%)	303 (61.8%)	67 (26.9%)	91 (23.8%)	
23.0-24.9	700 (55.7%)	39 (28.9%)	187 (38.2%)	182(73.1%)	292 (76.2%)	
SBP (mmHg), *n* (%)						0.085
≤117	608 (48.4%)	57 (42.2%)	244 (49.8%)	116 (46.6%)	191 (49.9%)	
>117	649 (51.6%)	78 (57.8%)	246 (50.2%)	133 (53.4%)	192 (50.1%)	
DBP (mmHg), *n* (%)						0.972
≤71	614 (48.8%)	60 (44.4%)	240 (49.0%)	114 (45.8%)	200 (52.2%)	
>71	643 (51.2%)	75 (55.6%)	250 (51.0%)	135 (54.2%)	183 (47.8%)	
Alanine aminotransferase (U/L), *n* (%)						<0.001
≤17	448 (35.6%)	68 (50.4%)	253(51.6%)	49 (19.4%)	78 (20.4%)	
>17	809 (64.4%)	67 (49.6%)	237 (48.4%)	200 (80.3%)	305 (79.6%)	
Aspartate aminotransferase (U/L), *n* (%)						<0.001
≤18	500 (39.8%)	70 (51.9%)	256 (52.2%)	62 (24.9%)	112 (29.2%)	
>18	757 (60.2%)	65 (48.1%)	234 (47.8%)	187 (75.1%)	271 (70.8%)	
Total cholesterol (mmol/L), *n* (%)						0.174
≤ 4.71	592 (47.1%)	67 (49.6%)	247 (50.4%)	109 (43.8%)	169 (44.1%)	
>4.71	665 (52.9%)	68 (50.4%)	243 (49.6%)	140 (56.2%)	214 (55.9%)	
High-density lipoprotein cholesterol (mmol/L), *n* (%)						<0.001
≥1.36	560 (44.6%)	69 (51.1%)	249 (50.8%)	86 (34.5%)	156 (40.7%)	
<1.36	697 (55.4%)	66 (48.9%)	241 (49.2%)	163 (65.5%)	227 (59.3%)	
Triglyceride (mmol/L), *n* (%)						<0.001
≤0.9	425 (33.8%)	69 (51.1%)	249 (50.8%)	38 (15.3%)	69 (18.0%)	
>0.9	832 (66.2%)	66 (48.9%)	241 (49.2%)	211 (84.7%)	314 (82.0%)	
Low-density lipoprotein cholesterol (mmol/L), *n* (%)						0.031
≤2.65	585 (46.5%)	72 (53.3%)	244 (49.8%)	103 (41.4%)	166 (43.3%)	
>2.65	672 (53.5%)	63 (46.7%)	246 (50.2%)	146 (58.6%)	217 (56.7%)	
Glucose (mmol/L), *n* (%)						<0.001
≤5.25	628 (50.0%)	83 (61.5%)	375 (76.5%)	47 (18.9%)	123(32.1%)	
>5.25	629 (50.0%)	52 (38.5%)	115 (23.5%)	202 (81.1%)	260 (67.9%)	
Glycated hemoglobin (%), *n* (%)						<0.001
≤5.5	543 (43.2%)	86 (63.7%)	273 (55.7%)	50 (20.1%)	134 (35.0%)	
>5.5	714 (56.8%)	49 (36.3%)	217 (44.3%)	199 (79.9%)	249 (65.0%)	
Fasting insulin (uIU/ml), *n* (%)						<0.001
≤3.89	915 (72.8%)	85 (63%)	429 (87.6%)	62 (24.9%)	339 (88.5%)	
>3.89	342 (27.2%)	50 (37%)	61 (12.4%)	187 (75.1%)	44 (11.5%)	
Homeostasis model assessment-beta (%), *n* (%)						<0.001
≥47.1	506 (40.3%)	119 (88.1%)	220 (44.9%)	107 (43.0%)	60 (15.7%)	
<47.1	751 (59.7%)	16 (11.9%)	270 (55.1%)	142 (57.0%)	323 (84.3%)	

**Table 2:** Factors associated with the presence of nonobese NAFLD in the HOMA-IR >1 group.

HOMA − IR > 1	Model 1		Model 2		Model 3	
Model variables	OR (95% CI)	*P* value	OR (95% CI)	*P* value	OR (95% CI)	*P* value
Gender						
Female	1.00		—	—	—	—
Male	1.261 (0.801-1.987)	0.317	—	—	—	—
Age (years)						
18-30	1.00		1.00		1.00	
31-40	1.064 (0.567-1.994)	0.847	1.064 (0.567-1.994)	0.847	1.118 (0.425-2.938)	0.821
41-50	1.715 (0.903-3.256)	0.099	1.715 (0.903-3.256)	0.099	2.492 (0.919-6.759)	0.073
>50	2.082 (1.105-3.923)	0.023	2.082 (1.105-3.923)	0.023	3.253 (1.224-8.647)	0.018
Body mass index (kg/m^2^)						
18.5-22.9	1.00		1.00		1.00	
23.0-24.9	6.687 (4.198-10.653)	<0.001	6.687 (4.198-10.653)	<0.001	5.532 (2.830-10.813)	<0.001
Alanine aminotransferase (U/L)						
≤17	1.00				—	—
>17	4.143 (2.616-6.561)	<0.001	4.525 (2.811-7.285)	<0.001	—	—
Aspartate aminotransferase (U/L)						
≤18	1.00		1.00		1.00	
>18	2.248 (2.085-5.060)	<0.001	3.458 (2.194-5.451)	<0.001	2.326 (1.192-4.539)	0.013
Total cholesterol (mmol/L)						
≤4.71	1.00		—	—	—	—
>4.71	1.266 (0.831-1.926)	0.272	—	—	—	—
High-density lipoprotein cholesterol (mmol/L)						
≥1.36	1.00		1.00		—	—
<1.36	1.982 (1.293-3.036)	0.002	1.982 (1.293-3.036)	0.002	—	—
Triglyceride (mmol/L)						
≤0.9	1.00		1.00		1.00	
>0.9	5.805 (3.582-9.408)	<0.001	6.017 (3.677-9.846)	<0.001	6.069 (2.899-12.708)	<0.001
Low-density lipoprotein cholesterol (mmol/L)						
≤2.65	1.00		1.00		—	—
>2.65	1.620 (1.062-2.470)	0.025	1.636 (1.066-2.510)	0.024	—	—
Glucose (mmol/L)						
≤5.25	1.00		1.00		1.00	
>5.25	6.860 (4.288-10.976)	<0.001	7.400 (4.555-12.022)	<0.001	2.953 (1.376-6.339)	<0.001
Homeostasis model assessment-beta (%)						
≤5.5	1.00		1.00		1.00	
>5.5	6.985 (4.374-11.156)	<0.001	6.985 (4.374-11.156)	<0.001	5.434 (2.765-10.682)	<0.001
Fasting insulin (uIU/mL)						
≤3.89	1.00		1.00		1.00	
>3.89	5.127 (3.262-8.059)	<0.001	5.280 (3.328-8.378)	<0.001	2.890 (1.492-5.598)	0.002
Homeostasis model assessment-beta (%)						
≥47.1	1.00		1.00		1.00	
<47.1	9.870 (5.532-17.612)	<0.001	12.194 (6.643-22.381)	<0.001	7.460 (3.051-18.238)	<0.001

Abbreviations: 95% CI: 95% confidence interval; OR: odds ratio; †: the presence or absence of NAFLD was the dependent variable. Model 1: not adjusted for any confounding factors. Model 2: adjusted for gender and age. Model 3: adjusted for variables included in the model such as gender; age (years) 31 to 40, 41 to 50, and >50; BMI (kg/m^2^) 23.0 to 24.9; ALT > 17 U/L; AST > 18 U/L; TC > 4.71 mmol/L; HDL − c < 1.36 mmol/L; TG > 0.9 mmol/L; LDL − c > 2.65 mmol/L; GLU > 5.25 mmol/L; HbA1C > 5.5%; and HOMA − *β* < 47.1%.

**Table 3:** Factors associated with the presence of nonobese NAFLD in the HOMA − IR ≤ 1 group.

HOMA − IR ≤ 1	Model 1		Model 2		Model 3	
Variables	OR (95% CI)	*P* value	OR (95% CI)	*P* value	OR (95% CI)	*P* value
Gender						
Female	1.00		1.00		—	—
Male	1.431 (1.066-1.920)	0.017	1.525 (1.124-2.071)	0.007	—	—
Age (years)						
18-30	1.00		1.00		1.00	
31-40	1.014 (0.668-1.620)	0.860	1.076 (0.689-1.678)	0.748	1.539 (0.871-2.717)	0.138
41-50	2.684 (1.753-4.108)	<0.001	2.748 (1.791-4.216)	<0.001	2.993 (1.744-5.137)	<0.001
>50	3.104 (2.035-4.736)	<0.001	3.270 (2.135-5.008)	<0.001	3.086 (1.810-5.261)	<0.001
Body mass index (kg/m^2^)						
18.5-22.9	1.00		1.00		1.00	
23.0-24.9	5.199 (3.861-7.002)	<0.001	4.996 (3.682-6.777)	<0.001	4.522 (3.104-6.588)	<0.001
Alanine aminotransferase (U/L)						
≤17	1.00				—	—
>17	4.174 (3.076-5.665)	<0.001	4.044 (2.955-5.535)	<0.001	—	—
Aspartate aminotransferase (U/L)						
≤18	1.00		1.00		1.00	
>18	2.647 (1.995-3.512)	<0.001	2.553 (1.903-3.425)	<0.001	1.778 (1.231-2.569)	<0.001
Total cholesterol (mmol/L)						
≤4.71	1.00		—	—	—	—
>4.71	1.287 (0.984-1.683)	0.065	—	—	—	—
High-density lipoprotein cholesterol (mmol/L)						
≥1.36	1.00		1.00		—	—
<1.36	1.503 (1.148-1.969)	0.003	1.445 (1.087-1.921)	<0.001	—	—
Triglyceride (mmol/L)						
≤0.9	1.00		1.00		1.00	
>0.9	4.702 (3.431-6.443)	<0.001	4.783 (3.452-6.627)	<0.001	3.423 (2.429-4.823)	<0.001
Low-density lipoprotein cholesterol (mmol/L)						
≤2.65	1.00		—	—	—	—
>2.65	1.297 (0.991-1.696)	0.058	—	—	—	—
Glucose (mmol/L)						
≤5.25	1.00		1.00		1.00	
>5.25	6.893 (5.109-9.299)	<0.001	6.798 (4.982-9.276)	<0.001	7.168 (4.935-10.409)	<0.001
Glycated hemoglobin (%)						
≤5.5	1.00		1.00		1.00	
>5.5	4.922 (3.669-6.602)	<0.001	4.691 (3.467-6.348)	<0.001	4.757 (2.601-5.429)	<0.001
Fasting insulin (uIU/mL)						
≤3.89	1.00		—	—	—	—
>3.89	0.913 (0.604-1.380)	0.665	—	—	—	—
Homeostasis model assessment-beta (%)						
≥47.1	1.00		1.00		—	
<47.1	4.386 (3.160-6.089)	<0.001	4.261 (3.039-5.974)	<0.001	—	—

Abbreviations: 95% CI: 95% confidence interval; OR: odds ratio; †: the presence or absence of NAFLD was the dependent variable. Model 1: not adjusted for any confounding factors. Model 2: adjusted for gender and age. Model 3: adjusted for variables included in the model such as gender; age (years) 31 to 40, 41 to 50, and >50; BMI (kg/m^2^) 23.0 to 24.9; ALT > 17 U/L; AST > 18 U/L; TC > 4.71 mmol/L; HDL − c < 1.36 mmol/L; TG > 0.9 mmol/L; LDL − c > 2.65 mmol/L; GLU > 5.25 mmol/L; HbA1C > 5.5%; and HOMA − *β* < 47.1%.

## Data Availability

The data used to support the findings of this study are available from the corresponding authors upon request.
